# Multiplexed Remote SPR Detection of Biological Interactions through Optical Fiber Bundles

**DOI:** 10.3390/s20020511

**Published:** 2020-01-16

**Authors:** Cloé Desmet, Karim Vindas, Ricardo Alvarado Meza, Patrick Garrigue, Silvia Voci, Neso Sojic, Ali Maziz, Rémi Courson, Laurent Malaquin, Thierry Leichle, Arnaud Buhot, Yoann Roupioz, Loic Leroy, Elodie Engel

**Affiliations:** 1 Univ. Grenoble Alpes, CEA, CNRS, SyMMES, 38000 Grenoble, France; cloe.desmet@ec.europa.eu (C.D.); karim.vindas@gmail.com (K.V.); ricardoalvarado@gmail.com (R.A.M.); arnaud.buhot@cea.fr (A.B.); yoann.roupioz@cea.fr (Y.R.); loic.leroy@univ-grenoble-alpes.fr (L.L.); 2 Univ. Bordeaux, INP-Bordeaux, ISM, CNRS UMR5255, 33607 Pessac, France; patrick.garrigue@enscbp.fr (P.G.); silvia.voci@enscbp.fr (S.V.); neso.sojic@enscbp.fr (N.S.); 3 CNRS, LAAS, 7 avenue du colonel Roche, F-31400 Toulouse, France; amaziz@laas.fr (A.M.); remi.courson@laas.fr (R.C.); laurent.malaquin@laas.fr (L.M.); thierry.leichle@laas.fr (T.L.)

**Keywords:** optical fiber, biosensor, surface plasmon resonance, SPR, label-free, multiplexed detection, biomolecular detection, functionalization, microstructuration

## Abstract

The development of sensitive methods for in situ detection of biomarkers is a real challenge to bring medical diagnosis a step forward. The proof-of-concept of a remote multiplexed biomolecular interaction detection through a plasmonic optical fiber bundle is demonstrated here. The strategy relies on a fiber optic biosensor designed from a 300 µm diameter bundle composed of 6000 individual optical fibers. When appropriately etched and metallized, each optical fiber exhibits specific plasmonic properties. The surface plasmon resonance phenomenon occurring at the surface of each fiber enables to measure biomolecular interactions, through the changes of the retro-reflected light intensity due to light/plasmon coupling variations. The functionalization of the microstructured bundle by multiple protein probes was performed using new polymeric 3D-printed microcantilevers. Such soft cantilevers allow for immobilizing the probes in micro spots, without damaging the optical microstructures nor the gold layer. We show here the potential of this device to perform the multiplexed detection of two different antibodies with limits of detection down to a few tenths of nanomoles per liter. This tool, adapted for multiparametric, real-time, and label free monitoring is minimally invasive and could then provide a useful platform for in vivo targeted molecular analysis.

## 1. Introduction

The increase of life expectancy is driving the need for innovative and affordable means to maintain a good health among the ageing population. Medical devices, point-of-care and diagnosis methods are therefore one of the hot topics in scientific research, closely associated with the advances in bio-and nano-technologies. While optical fibers are mostly known for their use in telecommunication since they permit transferring large data volumes over long distances in short amounts of time, their development as fiberscopes has opened opportunities for a wide range of fields, to examine difficult-to-reach areas. For instance, optical fibers have been used in medicine for imaging purposes by endoscopy for almost 70 years, enabling to precisely target an organ and to move inside the body [1]. Particularly attractive for medical diagnosis, they started to be developed as medical sensors since the 1970s [2,3,4,5,6]. The first main applications with sensing purposes focused on pH and temperature measurements but flow, pressure or blood gas analysis have also been described [7,8,9,10]. These particular remote sensors have a number of advantages compared to most ex situ optical sensors. They can be introduced into living tissues with very little damage and remote measurement sites can be reached. Thus, they enable avoiding the collection of samples to be investigated in equipped laboratories, providing the possibility to overcome the drawbacks of ex situ biomarker measurement techniques which are invasive, time-consuming, and expensive. Moreover, this targeting capacity would permit to reach specific microenvironments where target biomolecules are present in higher concentration (tumors [11,12] or lymph nodes [13,14] for instance), for a better chance of early detection. 

Biomolecular optical fiber sensing is based on different processing methods, frequently relying on the interaction of the evanescent field with the external medium. Indeed, as label free detection can be source of significant advances to produce tools for rapid medical diagnosis, a great number of approaches based on surface plasmon resonance (SPR) phenomenon have been developed [15,16,17,18,19,20,21]. Several strategies exist to enable the evanescent field to reach the sensed medium: cladding removed evanescent wave, and tapered or fiber Bragg grating configurations were mainly described. Most applications described so far only permit analyzing one parameter, or one biomarker, at a time [22,23,24], although only few examples describe the use of a single optical fiber for multiplex analysis. Sciacca et al. performed a double in vitro detection on a single optical fiber using two types of nanoparticles (gold and silver) [25]. Verma et al. have associated three sensitive areas, each covered with a different metal, in series on the same fiber [26]. Despite the progress made, these approaches, by their very principle of operation, are inherently limited to only a small multiplexing (2 to 3 analyses), are not easily adaptable to in situ measurement and require complex techniques for optical fiber fabrication. The ability to perform a remote multiplexed in situ detection remains an important challenge in the case of medical diagnosis. Different measuring areas are required for applications in complex media (as blood, serum, etc.) to include negative controls, necessary to correct the signal from unspecific contributions. Moreover, the research on microarrays and biosensing technologies for in vitro diagnosis is driven by the need for multiparametric analysis, applied to biomarkers detection and quantification for instance [5,27,28,29,30]. Gathering together optical fibers and microarrays to build a remote multiparametric optical fiber biosensor [5,29,31] would make possible targeted bioanalysis, representing a real asset in numerous cases such as monitoring organ-specific biomarkers.

In a recent report [32], our team has demonstrated for the first time highly-parallel remote SPR detection of DNA hybridization via a microstructured optical fiber bundle. Nevertheless, the functionalization of a non-planar surface such as this bundle presented a challenge [33,34,35,36,37,38] because of the lack of an adapted multi-functionalization process. We were able to functionalize the whole surface with a single probe and thus to detect only one DNA target. In the present paper, we first demonstrate our ability to graft two different probes and keep a control area onto the same bundle by means of a suitable micro-scaled biofunctionalization tool. Secondly, we validate the proof of concept of the detection of two different targets under model conditions. Finally, we show the full potential of the obtained miniaturized biosensor by monitoring remotely and in a label-free manner the multiplexed detection of two antibodies with detection limits in the order of a few tenth of nanomoles per liter. Both the functionalization and detection were also validated with fluorescence labeled molecules as a complementary method.

## 2. Materials and Methods

### 2.1. Reagents

Phosphate Buffer Saline (PBS), PEG_2000_SH, rabbit IgG, rat IgG, anti-rabbit-biotin IgG, anti-rat-biotin IgG, H_2_SO_4_, H_2_O_2_, Bovine Serum Albumin (BSA), Ethanol, Tween20 and glycerol were purchased from Sigma Aldrich (Saint Quentin Fallavier, France). Fluorescence validation was performed with Streptavidin-Phycoerythrin (SAPE) from Fisher Scientific (Illkirch, France). 

### 2.2. Optical Fiber Bundle Preparation

Silica imaging fibers with a diameter of 270 µm comprising 6,000 individually cladded 3–4 µm diameter optical fibers (Sumitomo Electric Industries, IGN-035/06, Osaka, Japan) were microstructured and gold coated to form arrays of micropillars exhibiting plasmonic properties (see Supplementary Materials Figure S1 for the principle of plasmon excitation), following a protocol specifically optimized as described elsewhere [32]. Briefly, the optical fiber bundle was cleaved and the distal face was then etched for 35 min in a buffer solution consisting of saturated NH_4_F and 48% of hydrofluoric acid HF (hydrogen fluoride) in proportion 5/1. (Caution: HF etching solutions are extremely corrosive and dangerous for health, safety procedures must be followed accordingly). The wet etching permitted to produce micropillars on each fiber, with base diameters of 2–3 µm, height of 7–10 µm, and half apex angle α of 10°, as measured on scanning electron microscopy images of the fibers (Supplementary Materials Figure S2). 

After a thorough cleaning of the bundle with ethanol and 24 s of plasma etching, the metallization process was realized using an electron gun sputter-coater with a specific support allowing the tilt of the sample for homogenization of the deposited films. The thickness of the different layers was measured using the quartz microbalance incorporated into the sputter-coater. In addition, 10 nm of titanium were first deposited as an adhesion layer, followed by 290 nm of gold ($Au_{flat}$) on the top of the micropillars. The resulting gold layer on the lateral surfaces ($Au_{side}$) then presents a thickness of 50 nm, as calculated from $Au_{side}=\;Au_{flat}\times \sin\left(\alpha \right)$ (see Supplementary Materials Figure S3 for details on the lateral gold thickness estimation).

### 2.3. Optical Setup and Characterization

An optical setup equipped with a 625nm LED source and a CMOS Camera (ORCA 4.0 LTE, Hamamatsu, Japan) was conceived as described in our previous study [32] to image and quantify the retro-reflected light coming back from the sensitive surface. This allowed the optical sensitivity characterization of the bundles, the monitoring of drop deposition on the different fibers composing the bundle but also to follow by surface plasmon resonance (SPR) any further modification occurring within the sensitive areas of the surface. The retro-reflected intensity can indeed be measured in real time on every fiber composing the bundle. 

Prior to any further modification or use of the system as a biosensor, the global sensitivity to refractive index changes of the bundle was characterized. The fiber gold-coated end-face was placed successively into solutions of known refractive indexes (deionized water (R.I = 1.332), PBS (R.I = 1.3364), glycerol 5% (R.I = 1.3421)), and several images were registered. The retro-reflected intensity I(n) was measured for the three solutions and their corresponding refractive indexes n. i(n), the relative normalized retro-reflected intensity, is defined as follows: $i\left(n\right)\;=\;\left(I\left(n\right)-I_{ref}\right)/I_{ref}$ where $I_{ref}$ represents the retro-reflected intensity in water. The sensitivity S, defined as the slope of the relative normalized retro-reflected intensity per Refractive Index Unit (RIU) $\frac{di}{dn}$, was quantified.

### 2.4. Surface Biofunctionalization

Prior to biofunctionalization, the microstructured gold coated surface was cleaned by immersion into a Piranha solution freshly prepared with H_2_SO_4_:H_2_O_2_ (3:1) for 1 min (Caution: The Piranha cleaning reaction is highly exothermic and extremely reactive, dedicated safety procedures must be followed accordingly). The surface was then thoroughly rinsed with deionized water (18 MΩ·cm) and left overnight to stabilize the surface reactivity and to obtain reproducible functionalization conditions.

A soft microcantilever (Bioplume V6, LAAS, Toulouse, France), a polymeric replication of a previously developed silicon microcantilever [39,40] fabricated by 3D-printing of a DS-3000 photoresist (DWS, Thiene, Italy) with a Dilase 3D printer (Kloe SA, St Mathieu de Tréviers, France) [41,42], was used to deposit microdrops of solutions on the microstructured face of the bundle. Details on the polymeric microcantilever conception are reported in the electronic Supplementary Materials (Figure S4). The cantilever was mounted together with a CMOS camera (DigiMicro 2.0 Scale, Toolcraft, Conrad, Haubourdin, France) on a computer-controlled *x*–*y* stage of 5 µm step precision and a manually controlled *z*-axis of micrometric precision. Different unmodified antibodies solutions at a concentration of 1 µM were used to functionalize the sensitive face of the sensor by physical adsorption as follows. A first probe solution was loaded on the microcantilever by immersion, a drop was deposited on flat glass in order to ensure proper deposition before moving the microcantilever on a localized *x*, *y* position on top of the bundle. A microdrop was then deposited on the microstructured face of the bundle, covering an area of around 200 micropillars (roughly corresponding to 2000 µm^2^ coated area, or around 1/20 of the total surface). The microcantilever was thoroughly washed with ethanol and water before loading the second probe solution for subsequent deposition. The spotted surface was left to react for at least 30 min for the protein immobilization by self-adsorption as a compact layer of non-oriented molecules on the gold cleaned surface. The whole surface was then rinsed by immersion in PBS and unfunctionalized areas were blocked for 30 min using a solution of PBS containing 1% BSA (*w*/*v*) in order to prevent non-specific adsorption at latter stage.

### 2.5. Multiplexed Biodetection Assays

The biofunctionalized surface was successively immersed in different Eppendorf microtubes containing target solutions, in concentration ranging from 0.1 nmol·L^−1^ to 10 µmol·L^−1^. Each solution was incubated for 15 min in order to reach signal stabilization before rinsing in a microtube of PBS during 3 min. The SPR signal was monitored by the optical system during the whole experiment by measuring the retro-reflected light intensity I(t) on the different spot localization as a function of time. The initial signal on each spot area, at the beginning of the experiment $t_{0}$ (before incubation of the target solutions), was reduced to zero by subtraction of the mean intensity on the area at $t_{0}$, $I\left(t_{0}\right)$. The mean intensity on a negative control area of an equal surface $\left(I_{neg}\left(t\right)-\;I_{neg}\left(t_{0}\right)\right)$ was subtracted to the previous signal all along the experiment in order to take into account intensity variations induced by unspecific phenomena. The exploited signal i(t) is finally given by:

(1) $$i\left(t\right)=\left(I\left(t\right)-I\left(t_{0}\right)\right)-\left(I_{neg}\left(t\right)-\;I_{neg}\left(t_{0}\right)\right)$$

In order to confirm the multiplexed biodetection observed by SPR by an independent method, biotinylated antibodies were used and revealed by SPR and fluorescence after subsequent incubation in streptavidin-R-phycoerythrin (10% *v*/*v* in PBS) for 15 min and rinsing with PBS-Tween20 (0.05% *v*/*v*) in order to remove non-specifically bound molecules. Fluorescence images of the reactive face of the bundle were acquired using a Leica DMI4000B (Leica Microsystemes SAS, Nanterre, France) inverted microscope.

## 3. Results and Discussion

### 3.1. Optical Setup and Characterization

The optical setup enabled to both inject light by the cleaved face of the optical fiber bundle and to image the retro-reflected light on the same side. The excitation light is guided by total internal reflection in the individual fiber cores through the bundle up to the microstructured surface (i.e., distal face) where it is confined in each core and senses the local optical index. The retro-reflected fraction of the light is collected by the same core and transmitted through to reach back the cleaved face. Each core of the bundle corresponds then to a single SPR sensor whose response was monitored by the camera. The preparation process of the optical fiber bundle, composed of the wet-etching microstructuration and gold coating, was controlled before the biodetection experiment by the characterization to the global refractive index change. Three solutions of different refractive indexes were used to assess the global sensitivity of the bundle. The sensitivity S, i.e., the intensity change by refractive index unit, was evaluated here as −250%/RIU for the whole fiber bundle. As reported in our previous study [32], this sensitivity corresponds to a resolution (i.e., the smallest detectable optical index variation) in the order of $10^{–4}$ refractive index unit (RIU). These sensitivity and resolution values validate the different bundle preparation steps (etching, gold coating) and are suitable with the measurement of bimolecular interactions [32].

### 3.2. Surface Biofunctionalization

The gold-coated surface of the optical fiber bundle was used as a transducer platform to perform the biosensing assays. While the geometry of the microstructured optical fiber bundles gives them plasmonic properties and their size makes them interesting for in vivo sensing, these two aspects also add complexity to the functionalization process. We previously described the immobilization of a single probe on the surface of an optical fiber bundle and the detection of the corresponding target by around 80% of the optical fibers of the bundle [32]. However, this sensor being sensitive to intensity changes due to unspecific phenomena, direct implementation for biomedical application would be problematic. In order to consider any change of the sensed media non-correlated with the presence of a target molecule, different sensing areas must be defined at the sensor surface. The multiple functionalization of the surface will permit differentiating the signal given by an unspecific phenomenon (bulk change of refractive index or unspecific surface interactions) from the binding of a specific target. Moreover, it is now widely accepted that detecting a combination of biomarkers provides a mean of improving sensitivity and specificity for both diagnostic and prognostic agendas for a very large range of diseases [43,44,45,46]. 

**Figure 1:**
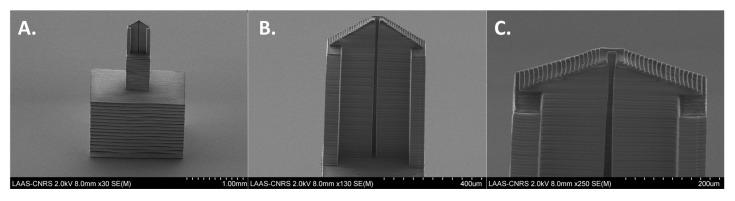
Scanning Electron Microscopy (SEM) pictures of a polymeric microcantilever used to perform the antibodies immobilization at different magnifications. (See electronic Supplementary Materials for more information on its conception).

**Figure 2:**
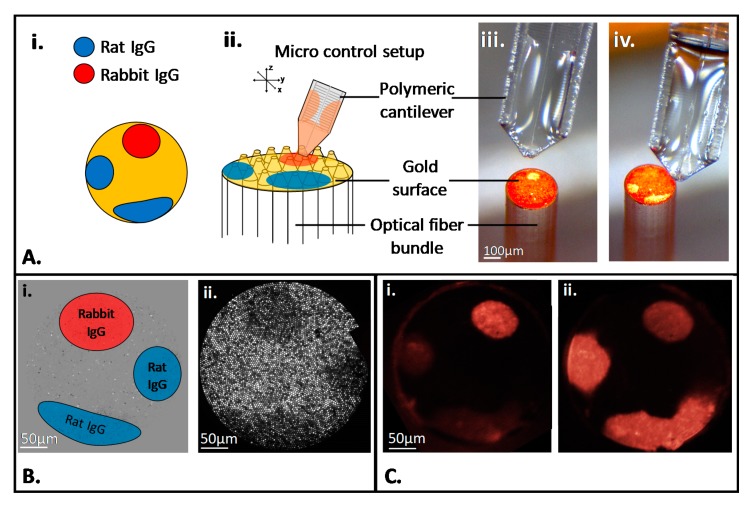
(**A**) multifunctionalization of the microstructured gold coated surface according to i. Spotting map, and ii. Scheme of the deposition, with the image corresponding to iii. Rabbit-IgG spot formation, iv. Rat IgG spots formation. Insert in (**A**) ii. Scanning Electron Microscopy (SEM) image of the microstructured gold coated surface. (**B**). Surface Plasmon Resonance (SPR) view of the droplets deposition with i. Spotting map on subtracted image, ii. Image of the retro-reflected light; (**C**) confirmation of the immobilization and antibodies detection by fluorescence microscopy with streptavidin-Phycoerythrin after addition of the biotinylated antibodies: i. Anti-rabbit and ii. Anti-rat.

Different methods were tested to immobilize multiple probes on the microstructured surface of the bundle. Among them, micro-contact deposition using soft microcantilevers was the most well-suited due to the technical complexity associated with the handling of the optical fiber bundle microstructured face. Previously used silicon cantilevers for optical fiber functionalization [35] did not allow a proper deposition of liquid drops onto the etched fibers used in this work as they damaged the higher aspect ratio and more fragile micropillar structures (See electronic Supplementary Materials Figure S5). Newly developed 3D-printed polymeric microcantilevers [41,42] (Figure 1 and electronic Supplementary Materials Figure S4) were soft enough to touch the apex without breaking the structures nor damaging the gold layer, and to deposit microdrops of solution with an appropriate size (see Figure 2A for the droplets’ deposition plan (i), the schematic deposition principle (ii) and the images of the droplets deposition with the soft polymeric cantilever (iii, iv)). The spotting solutions were prepared with 5% glycerol in order to make them more viscous for liquid deposition and to avoid a complete spot drying. The spotting process was monitored in real time by the optical setup. The difference of the retro-reflected light intensity between the dry surfaces and the one in the deposited solution permitted first to validate the drops deposition when getting in contact with the surface, but also to localize the spots precisely for further analysis of the biomolecular interaction events at the surface (Figure 2B). To demonstrate the proof of concept, three spotted areas were realized on the surface; two different probes were used, a rabbit IgG, and a rat IgG; and one solution (rat IgG) was deposited in duplicate. The whole surface was finally blocked with BSA in order to reduce unspecific protein adsorption. The un-spotted domains of the bundle surface were used as a negative control area and could then be monitored to sense any global change at the surface, due to temperature or global refractive index variations or to unspecific surface interactions for instance. The resulting unspecific signal was then subtracted from the specific signal recorded on the spots. This step is necessary for the future use of the device for in vivo diagnosis since the refractive index of the environment can vary without being correlated to the presence of targeted molecules. Fluorescence was used as an alternative analytical method to confirm the functionalization of the fiber bundle by two different probes. Figure 2C demonstrates the specific immobilization and localization of the two different probes.

### 3.3. Multiplexed Biodetection Assays

**Figure 3:**
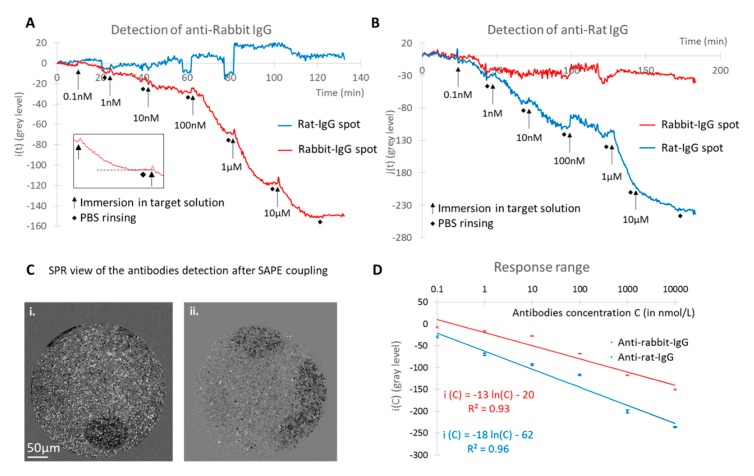
SPR signal (*i*(*t*), in gray level) monitoring on the different spots of the bundle for the detection of (**A**) anti-rabbit IgG and (**B**) anti-rat IgG; (**C**) Surface Plasmon Resonance (SPR) images of the light retro-reflected on the fibers after injection of i. anti-rabbit IgG and ii. anti-rat IgG followed by coupling with streptavidin-phycoerythrin. (**D**) response range of the SPR signal on the different spots as a function of the corresponding specific antibodies concentration. Insert in A. zoom in one concentration of the curve, showing signal stabilization and rinsing in Phosphate-Buffered Saline (PBS).

In order to demonstrate the feasibility of a multiplexed detection by SPR on the microstructured optical fiber bundle, and to confirm it afterwards by fluorescence microscopy, two anti-species antibodies labelled with a fluorophore were used as targets. A concentration range of a first antibody was incubated on the multifunctionalized surface of the fiber bundle. The bundle was exposed to each solution for 15 min, followed by PBS rinsing. The bundle was first immersed in the anti-rabbit IgG solutions, starting from a concentration of 0.1 nmol·L^−1^ and up to 10 µmol·L^−1^. The retro-reflected light of the whole bundle was monitored in real time as described in the previous paragraph. The mean intensities on the different spots were measured and the background intensity given by the un-spotted area was subtracted to obtain i(t) for each spot as decribed previously in the materials and methods section. The results of the signal analysis on the two spots are shown in Figure 3A,B.

As expected, a biomolecular interaction between the rabbit IgG and anti-rabbit IgG was observed by a change in the signal intensity on the corresponding spot while the signal variation occurring on the rat IgG spot was much lower, showing a low cross-reactivity between the antibodies. The rinsing steps led to the stabilization of the signal at each concentration, demonstrating that only the loosely bound molecules were washed off from the surface while the specific antibody-antigen interactions remained stable. Because of the large excess of immobilized probes compared to the targets concentration in solution, the signal obtained from one incubated concentration was not affected by the previous one. Moreover, the effect of the concentration range was clearly visible as an increase in the target concentration results in a negative increase in the measured signal for the corresponding spot only. Protein concentrations as low as 1 nmol·L^−1^ were specifically detected by SPR. Streptavidin labelled with a fluorophore was then incubated on the sensing surface in order to validate the specific detection not only by SPR but also by fluorescence microscopy. This step enabled to visualize the spot by fluorescence (Figure 2C) and to enhance the SPR signal so that it can be directly visualized as shown in Figure 3C. As previously mentioned, the fluorescence observed in Figure 2C validated the biofunctionalization of the microstructured surface, but it also confirmed the specific biomolecular interaction at the surface of the bundle and demonstrated the preservation of antibody functionality after arraying on the spotted fiber bundle. The same steps were implemented with anti-rat IgG and validated the potential of multiplexed detection with the device. Figure 3D represents the calibration curves corresponding to the signal variation for both detected antibodies as a function of their concentration. The signal i(C) corresponding to a given concentration C has been set to the plateau value reached at the equilibrium. Experimental data showed that the equilibrium response i(C) scales logarithmically with the concentration, as it is generally the case for a specific concentration range in immunoassays in vitro or dose–response studies. In order to permit a better interpretation of the data, the *x*-axis is then presented in logarithmic scale in Figure 3D. Error bars of calibration curves were evaluated for each concentration from the signal standard deviation σ observed on the plateau values in Figure 3A,B corresponding to the standard deviation between nine individual measurements at the same concentration. As observed in Figure 3D, the response to the logarithm of the concentration is linear i(C)=αln(C)+β for both antibodies: i(C)=−13ln(C)−20 for anti-rabbit and i(C)=−18ln(C)−62 for anti-rat. This linear relationship allows for determining the Limit Of Detection (LOD) as the lowest concentration leading to a signal equal to 3 times: i(LOD)=3σ.

LODs for the anti-rabbit IgG and the anti-rat IgG detection were respectively estimated to 0.4 nM and 0.1 nM. These detection limits, in the order of a few tenths of nanomoles per liter, demonstrate a good sensitivity of the lab-on-fiber and represent the first LODs measured for the detection of antibodies by a multiplexed plasmonic optical fiber sensor used in reflection configuration. While the antibodies used here are not clinically relevant, they permitted to demonstrate the proof of concept of this system for the multiplexed detection of antibodies. We can then envision to apply this system to the detection of clinically relevant targets, such as auto-antibodies biomarkers for cancer diagnosis or antibodies resulting from vaccination [46,47,48,49,50]. The application of the system in vivo will require an evaluation of the possible interferences since the measurements here performed in vitro present a non-realistic situation of biomolecules in a buffered environment without change in pH, temperature, etc. of the medium. The effects related to this kind of changes represent the most important possible limitation of the system, but could be monitored thanks to the different areas of the fiber. The stability of the surface could also represent a challenge in an environment composed of a large amount of biomolecules, which could decrease the sensitivity of the sensor. To improve the stability of the surface biofunctionalization, we will have to use more specific chemistry like thiol self-assembly on the gold surface, which lead to a chemically stable gold-sulphur bond. Even if thiol-chemistry is compatible with applications in complex media such as blood plasma or serum, we will have to evaluate the stability of the probe grafting in biological media. Moreover, as for any immunoassay, the cross-reactivity of different probes and targets will have to be evaluated according to the application, in order to ensure the accurate detection of biomarkers.

## 4. Conclusions

In this study, we have presented the development of a new type of optical biosensor on a 300 µm diameter microstructured plasmonic fiber bundle. This “lab-on-fiber” was then used as a multifunctionalized optical platform to validate the proof of concept of a multiplexed, remote and label free detection of two antibodies. A target concentration range study allowed us to determine limits of detection on the order of a few tenth of nanomoles per liter for both antibodies, which represents a first estimate of antibodies LOD on a multiplexed plasmonic optical fiber sensor used in reflection mode. Every step, from the fiber functionalization to the biomolecules detection, was monitored by SPR imaging of the bundle and was validated by an alternative fluorescence method. The demonstrated potential of the sensor to perform a double detection and its intrinsic ability to be used in specific microenvironments open the way towards in vivo diagnosis. Indeed, the association of the optical fiber capacity to reach specific organs or tissues, to the multiplexed sensing, which is required for applications in complex media to take into account unspecific contributions, is of great value for the field. Moreover, it is particularly interesting to have several biomolecules analyzed on a single device in order to establish a reliable diagnosis. The next steps to improve our SPR sensing approach will be to obtain microarrays of probes with a better spatial resolution and to perform detections in more complex media as 1/10th diluted blood or serum, in order to get closer to medical applications.

## Supplementary Materials

The following are available online at https://www.mdpi.com/1424-8220/20/2/511/s1, Figure S1: Schematic representation of the SPR phenomenon occurring in an individual optical fiber in the micropillar configuration. In the present study, micropillars with the following characteristics have been fabricated: base diameters d of 2–3 μm, height h of 7–10 μm, half apex angle α of 10°. Figure S2: Principle of measurement of the half apex angle α on a SEM image of a micropillar structured optical fiber bundle. Figure S3: Principle of estimation of the gold thickness on the lateral faces of the micropillars. Figure S4: STL file of the microcantilever. Total dimension of the sample is 20 × 10 × 3.5 mm^3^, Figure S5: Micropillar structures damages caused by previously used silicon cantilevers for optical fiber functionalization. (A) S.E.M image of Bioplume Si cantilever. (B) Optical image of spotting process with Si cantilever. (C) Retro reflected image of the functionalized fiber bundle. (D) S.E.M image of induced defects.
