# Supplementary material for: Multiplexed Remote SPR Detection of Biological Interactions through Optical Fiber Bundles
Source: Sensors (Basel). 2020 Jan 16;20(2):511. doi: 10.3390/s20020511 (PMC7014493; doi:10.3390/s20020511)
Supplement: Supplementary file 1 [file sensors-20-00511-s001.pdf]

Article

Multiplexed Remote SPR Detection of Biological Interactions through Optical Fiber Bundles

Cloé Desmet [1], Karim Vindas [1], Ricardo Alvarado Meza [1], Patrick Garrigue [2], Silvia Voci [2], Neso Sojic [2], Ali Maziz [3], Rémi Courson [3], Laurent Malaquin [3], Thierry Leichle [3], Arnaud Buhot [1], Yoann Roupioz [1], Loic Leroy [1] and Elodie Engel [1,*]

[1] Univ. Grenoble Alpes, CEA, CNRS, SyMMES, 38000 Grenoble, France; cloe.desmet@ec.europa.eu (C.D.); karim.vindas@gmail.com (K.V.); ricardoalvarado@gmail.com (R.A.); arnaud.buhot@cea.fr (A.B.); yoann.roupioz@cea.fr (R.Y.); loic.leroy@univ-grenoble-alpes.fr (L.L.)

[2] Univ. Bordeaux, INP-Bordeaux, ISM, CNRS UMR5255, 33607 Pessac, France; patrick.garrigue@enscbp.fr (P.G.); silvia.voci@enscbp.fr (S.V.); neso.sojic@enscbp.fr (N.S.)

[3] CNRS, LAAS, 7 avenue du colonel Roche, F-31400 Toulouse, France; amaziz@laas.fr (A.M.); remi.courson@laas.fr (R.C.); laurent.malaquin@laas.fr (L.M.); thierry.leichle@laas.fr (T.L.)

* Correspondence: elodie.engel@univ-grenoble-alpes.fr

Received: 19 December 2019; Accepted: 14 January 2020; Published: date

Electronic Supplementary Materials

S1. Illustration of the SPR Phenomenon in an Individual Microstructured Optical Fiber

Excitation light was injected onto the cleaved face of the optical fiber bundle and guided by internal reflection in the individual fiber cores through the bundle. The light reaching the microstructured face (micropillar) was confined to the etched core and senses the local optical index. A fraction of the light was retro-reflected and collected by the same core, transmitted, and eventually detected at the cleaved face.

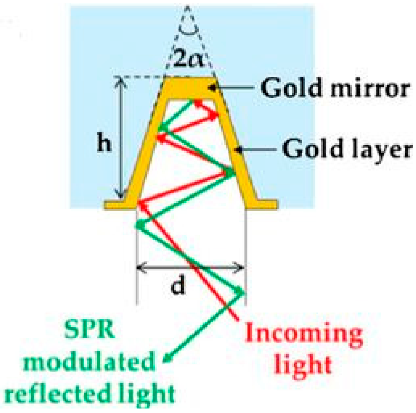

Figure S1. Schematic representation of the Surface Plasmon Resonance (SPR) phenomenon occurring in an individual optical fiber in the micropillar configuration. In the present study, micropillars with the following characteristics have been fabricated: base diameters d of 2–3 µm, height h of 7–10 µm, half apex angle α of 10°.

S2. Half Apex Angle Estimation

The estimation of the half apex angle was performed by analyzing scanning electron microscopy (SEM) images (Figure S2). To do this, we do rotate the sample until one of the flanks of the side face of the tip coincides with the observation axis. This gives a first angle ω_1. We then repeat the same measurement for the diametrically opposite flank which gives a second angle ω_2. The difference

$|\omega_1 - \omega_2|$ gives an estimate of 2α. The measurement principle is illustrated in Figure S2. This measurement provides an estimation of α to within one degree.

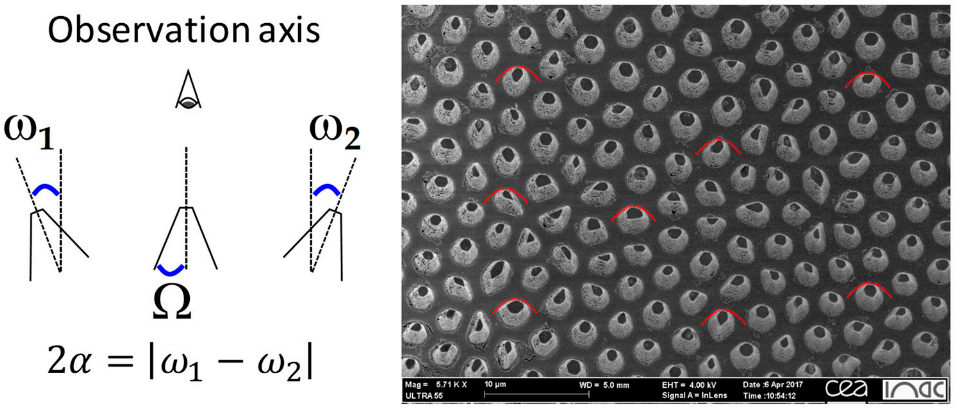

Figure S2. Principle of measurement of the half apex angle α on a Scanning Electron Microscopy (SEM) image of a micropillar structured optical fiber bundle.

S3. Gold Thickness Estimation

During the metallization process, the amount of gold deposited per length unit (along the x-axis, Figure S3) is considered constant. As a result, if the coated surface is tilted, the gold is distributed over a greater distance L and the thickness (Au_{side}) of the deposited layer is reduced compared to the thickness (Au_{flat}) deposited on a flat surface on a distance l. The amount of gold deposited on a flat surface, Au_{flat}, is measured with a quartz microbalance incorporated into the sputter-coated. We then obtain the coverage of the side, Au_{side}, that we can calculate from the half apex angle α and the gold amount measured on the flat surface: $Au_{side} = Au_{flat} \times \sin(\alpha)$, with Au_{flat} = 290 nm and a half apex angle α of 10°, we obtain Au_{side}= 50 nm.

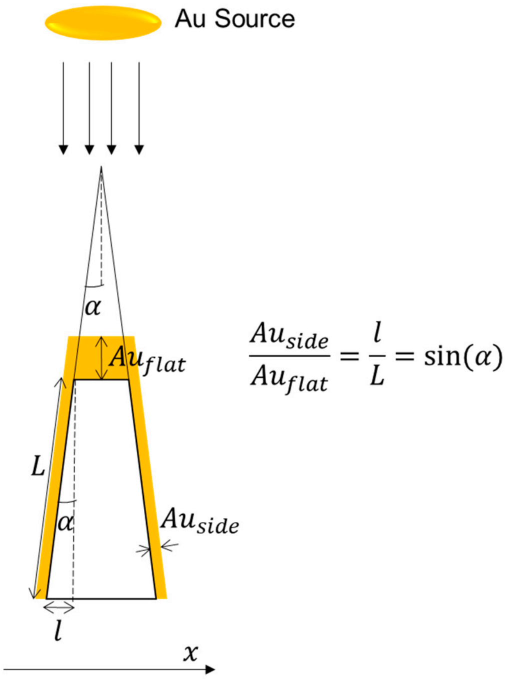

Figure S3. Principle of estimation of the gold thickness on the lateral faces of the micropillars.

S4. Polymeric Microcantilever Conception

A model of the cantilever to print is obtained with a CAO 3D software (leading to a Standard Tessellation Language file). Figure S4 shows a print screen of the schematic microcantilever. The most difficult parts to print are the 20 µm width × 50 µm depth × 1 mm long microchannel and the 60 × 60 μm^2 top footprint of the microcantilever. In the end, total dimension of the sample is 20 × 10 × 3.5 mm^3.

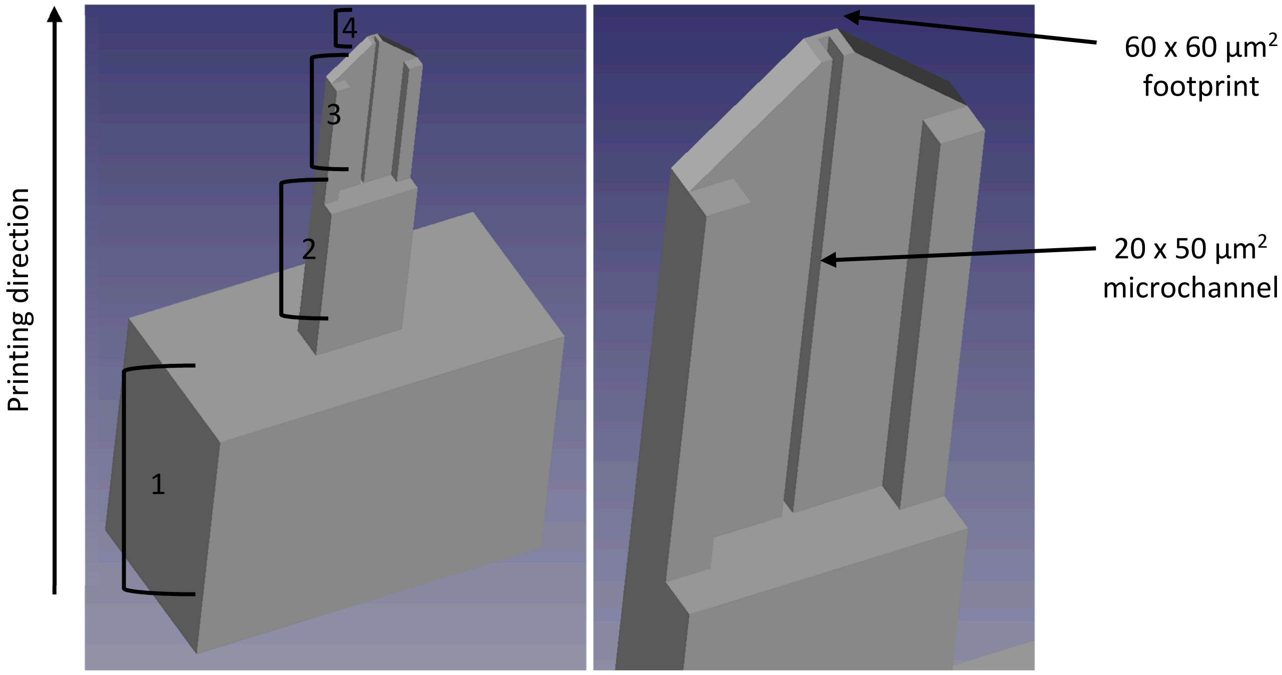

Figure S4. Stereolithography (STL) file of the microcantilever. Total dimensions of the sample are 20 × 10 × 3.5 mm^3.

Fabrication was conducted using a Dilase 3D printer (Kloe SA, St Mathieu de Tréviers, France), which was already described in our previous work [1].

Cantilevers were built directly onto a glass or silicon substrates with DS-3000 photoresist (DWS, Thiene, Italy). The first layer was fabricated at 100% laser power and 20 mm/s writing speed to ensure the adhesion onto the substrate. Then, for each region (increasing z, see Figure S4), applied parameters (slice size, laser power and writing speed) can be summarized as follows:

1. 0 to 1.5 mm: slice of 100 µm with 90% laser power and 30 mm/s.
2. 1.5 mm to 2.5 mm: slice of 50 µm with 90% laser power and 30 mm/s.
3. 2.5 mm to 3.3 mm: slice of 20 µm with 35% laser power and 6 mm/s.
4. 3.3 mm to 3.5 mm: slice of 10 µm with 35% laser power and 5 mm/s.

Total fabrication time for this model of cantilever was 3 h and 30 min. After laser writing, samples were removed from the build table and immersed into two successive baths filled with alcohol. Finally, samples were dried with nitrogen and gently manually detached from the substrate.

SEM imaging was performed on samples by using a Hitachi S-4800 microscope (Hitachi, Krefeld, Germany) with an acceleration voltage of 2 kV. Figure 1 in the main text shows three pictures of a microcantilever made in DS-3000 at different magnifications.

S5. Illustration of Micropillar Structures Damages Caused by Silicon Cantilevers

As mentioned in the main text, previously used silicon cantilevers for optical fiber functionalization [2] did not allow a proper deposition of liquid drops onto the etched fibers used in this work as they damaged the higher aspect ratio and more fragile micropillar structures (Figure S5)

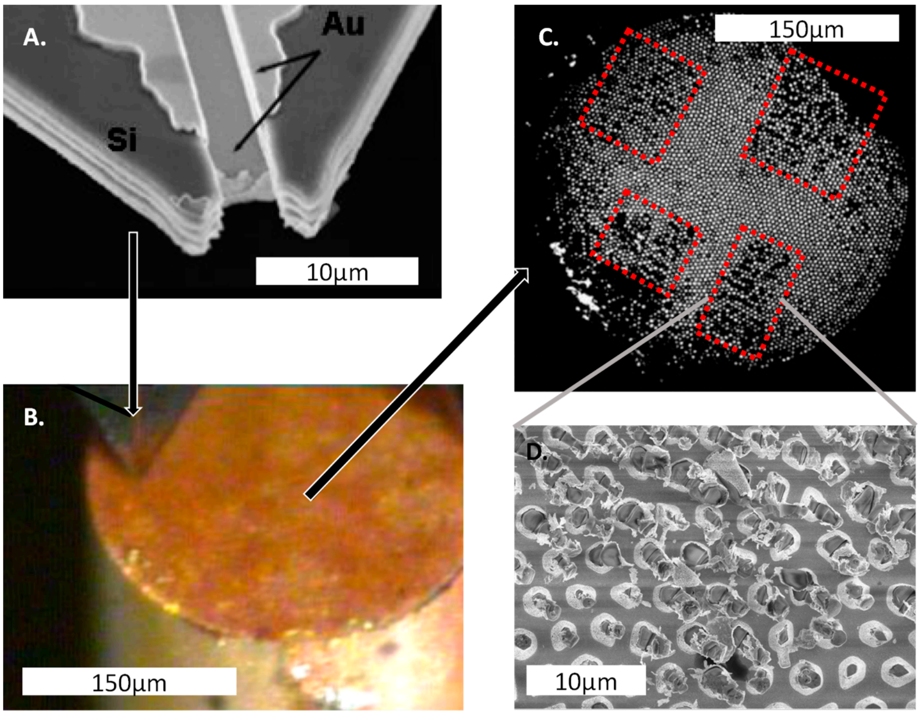

Figure S5. Micropillar structures damages caused by previously used silicon cantilevers for optical fiber functionalization. (**A**). S.E.M image of Bioplume Si cantilever; (B). optical image of spotting process with Si cantilever; (C). retro reflected image of the functionalized fiber bundle; (D). S.E.M image of induced defects.

References

1. Accardo, A.; Courson, R.; Riesco, R.; Raimbault, V.; Malaquin, L. Direct laser fabrication of meso-scale 2D and 3D architectures with micrometric feature resolution. *Addit. Manuf.* **2018**, *22*, 440–446.
2. Descamps, E.; Duroure, N.; Deiss, F.; Leichlé, T.; Adam, C.; Mailley, P.; Aït-Ikhlef, A.; Livache, T.; Nicu, L.; Sojic, N. Functionalization of optical nanotip arrays with an electrochemical microcantilever for multiplexed DNA detection. *Lab Chip* **2013**, *13*, 2956–2962.
